# *Sepia* Ink Oligopeptide Induces Apoptosis in Prostate Cancer Cell Lines via Caspase-3 Activation and Elevation of Bax/Bcl-2 Ratio

**DOI:** 10.3390/md10102153

**Published:** 2012-09-27

**Authors:** Fangfang Huang, Zuisu Yang, Di Yu, Jiabin Wang, Rong Li, Guofang Ding

**Affiliations:** 1 School of Food science and Pharmacy, Zhejiang Provincial Key Engineering Technology Research Center of Marine Biomedical Products, Zhejiang Ocean University, Zhoushan 316000, China; Email: gracegang@126.com (F.H.); abc1967@163.com (Z.Y.); yujiaxin1003@sina.com (D.Y.); saltrong0917@126.com (R.L.); 2 Zhijiang Hailisheng Group, Co., Ltd., Zhoushan 316000, China; Email: JiaBinWang@sina.cn

**Keywords:** oligopeptide, prostate cancer, apoptosis, proliferation

## Abstract

*Sepia* ink oligopeptide (SIO) is a tripeptide extracted from *Sepia* ink. To test the hypothesis that SIO inhibits prostate cancer by inducing apoptosis, the effects of SIO on the proliferation of three human prostate cancer cell lines were examined using a CCK-8 assay. SIO significantly inhibited the proliferation of DU-145, PC-3 and LNCaP cells in a time- and dose-dependent manner. Flow cytometry studies showed that exposing DU-145, PC-3 and LNCaP cells to 5, 10, or 15 mg/mL SIO for 24 h increased the percentage of the early-stage apoptotic cells from 11.84% to 38.26% (DU-145), 22.76% to 39.96% (PC-3) and 5.05% to 16.11% (LNCaP), respectively. In addition, typical morphologic changes were observed in the cells with acridine orange/ethidium bromide staining. SIO treatment induced strong S and G_2_/M phase cell cycle arrest in a dose-dependent manner in DU-145 and LNCaP. In contrast, SIO treatment induced strong Sub G_1_ and G_0_/G_1_ phase cell cycle arrest in a dose-dependent manner in PC-3. SIO exposure for 24 h decreased the expression of the anti-apoptotic protein Bcl-2 and increased the expression of the apoptogenic protein Bax. Moreover, the Bax/Bcl-2expression ratio was increased. Concurrently, the expression of caspase-3 was upregulated. These data support our hypothesis that SIO has anticarcinogenic properties.

## 1. Introduction

Prostate cancer is the second most frequently diagnosed cancer and the sixth leading cause of cancer death in men, accounting for 14% (903,500) of the total new cancer cases and 6% (258,400) of the total cancer deaths in men in 2008 [1]. Death rates from prostate cancer are decreasing in many developed countries, including Australia, Canada, the United Kingdom, the United States, Italy, and Norway, in part because of improved treatment with curative intent. In contrast to the trends in Western countries, however, the incidence and mortality rates are rising in several Asian and Central and Eastern European countries [2,3,4]. 

Treatment for prostate cancer may include active surveillance (monitoring for tumor progression or symptoms), surgery, radiation therapy, high-intensity focused ultrasound, chemotherapy, cryosurgery, hormonal therapy, or various combinations of these [5,6,7]. Chemotherapy is usually reserved for recurrent tumors that were previously treated with surgery and radiotherapy, or tumors for which surgery was not (or only partially) feasible, and for which the effect of radiotherapy is limited. Various chemotherapeutic regimens are used, most of which comprise a combination of drugs that are usually administered at high doses. Toxicity and drug resistance in patients are the main problems associated with standard chemotherapeutic regimens [8,9,10]. Therefore, natural antitumor agents for metastatic prostate cancer are urgently needed.

Newly discovered peptides are becoming increasingly important, not only as molecular tools for understanding protein-protein interactions, but also as lead compounds for the treatment of various diseases. Several studies have demonstrated that certain products from marine sources, such as *Cymbastella* sp., *Dolabella auricularia*, *Mactromeris polynyma*, and *Halichondria okadai*, have significant anticancer activity [11,12,13,14]. Dolastatins-10 and -15 are peptides isolated from the marine sea hare *Dolabella auricularia*, which have been known to have antitumor activities in several cancer cell lines [15,16].

*Sepia* ink oligopeptide (SIO), a tripeptide, is an anti-tumor peptide first isolated from *Sepia esculenta* by enzymolysis. *Sepia* ink possesses antitumor activity against Meth-A fibrosarcoma in BALB/c mice [17]. Peptides as antitumor drugs can improve immune responses, inhibit tumor angiogenesis and metastasis of tumor cells, directly eradicate tumor cells, induce tumor cell apoptosis and arrest the cell cycle [18]. Thus, SIO has a multitude of potential applications in human health care. Further, *Sepia* ink is in inexpensive commercial supply, as it is generally discarded during daily life and food processing. Our previous study [19] demonstrated that SIO significantly inhibits the proliferation of DU-145 cells and induces their death in a dose-dependent manner *in vitro*. The antitumor mechanisms of SIO against prostate cancer, however, are unclear.

In the present study, we evaluated the anticancer activity and related mechanisms of SIO against prostate cancer in cell culture. 

## 2. Results

### 2.1. SIO Inhibits Cell Proliferation of DU-145, PC-3 and LNCaP Cell Lines

**Figure 1:**
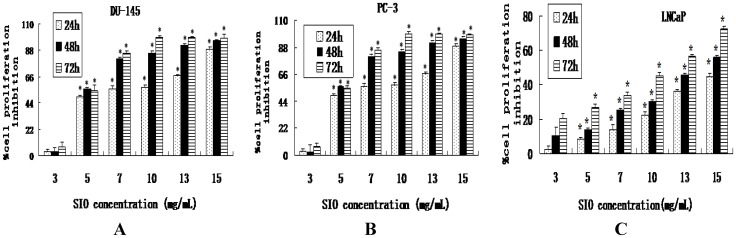
Inhibited proliferation of *Sepia* ink oligopeptide (SIO)-treated DU-145, PC-3 and LNCaP cells. (**A**) DU-145 cells were treated with 3, 5, 7, 10, 13 and 15 mg/mL SIO. (**B**) PC-3 cells were treated with 3, 5, 7, 10, 13 and 15 mg/mL SIO. (**C**) LNCaP cells were treated with 3, 5, 7, 10, 13 and 15 mg/mL SIO. Cell proliferation was measured using a CCK-8 assay at 24, 48, and 72 h after SIO treatment. SIO at doses of 5, 7, 10, 13, and 15 mg/mL significantly inhibited cell proliferation. Results are expressed as mean ± SD. Each experiment was performed in triplicate (*n* = 3). * Significant difference (*p* < 0.05) between treatments with the same concentration.

The anti-proliferative effects of SIO on human prostate cancer DU-145, PC-3 and LNCaP cells were first investigated by CCK-8 assay. SIO inhibited cell proliferation in a dose-and time-dependent manner, and the percent of inhibition of cell proliferation within 24 h after SIO treatment ranged from 10.58% to 74.62% in DU-145 cells (Figure 1A), 3.40% to 88.90% in PC-3 cells (Figure 1B) and 2.58% to 44.60% in LNCaP cells (Figure 1C), respectively. The inhibitory effect of SIO was significant at 24 h, and it was maximal at 48 h and 72 h. 

### 2.2. Morphologic Observation by Acridine Orange and Ethidium Bromide (AO/EB) Staining

**Figure 2:**
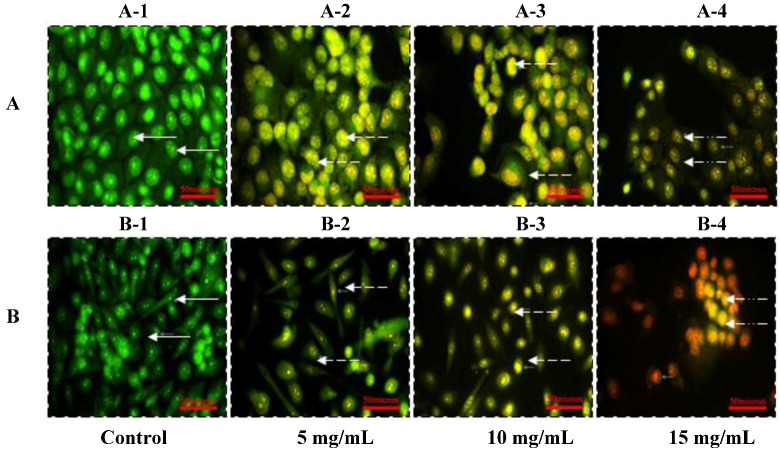
Morphologic observation with acridine orange/ethidium bromide (AO/EB) staining. DU-145 cells (**A**) were treated without (A-1) and with SIO at 5 mg/mL (A-2), 10 mg/mL (A-3), and 15mg/mL (A-4) for 24 h. PC-3 cells (**B**) were treated without (B-1) and with SIO at 5 mg/mL (B-2), 10 mg/mL (B-3), and 15 mg/mL (B-4) for 24 h. (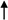) indicates viable cells; (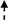) indicates early apoptotic cells; (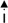) indicates late apoptotic cells. Each experiment was performed in triplicate (*n* = 3) and generated similar morphologic features. Original magnification 400×, bar = 50 μm.

To confirm that apoptosis was induced by SIO at concentrations of 5, 10, and 15 mg/mL, DU-145 and PC-3 cells were analyzed in the presence of acridine orange/ethidium bromide staining (AO/EB staining). As a control, DU-145 (Figure 2A-1) and PC-3 (Figure 2B-1) cells were cultured in F-12 media and stained with AO/EB. SIO at concentrations of 5, 10, and 15 mg/mL induced apoptosis after 24 h incubation (Figure 2). Cells stained green represent viable cells, whereas yellow staining represents early apoptotic cells, and reddish or orange staining represents late apoptotic cells. DU-145 cells treated with 5 mg/mL of SIO showed changes in cellular morphology, including chromatin condensation, membrane blebbing, and fragmented nuclei (Figure 2A). Similar features were observed in DU-145 and PC-3 cells treated with 10 mg/mL and 15 mg/mL SIO, but with additional features of late stage apoptotic activity with apoptotic bodies. AO/EB staining revealed that the morphologic features of apoptotic DU-145 and PC-3 cells were dose-dependent.

### 2.3. SIO Induces Apoptosis in DU-145, PC-3 and LNCaP Cells Based on Flow Cytometry Analysis

**Figure 3:**
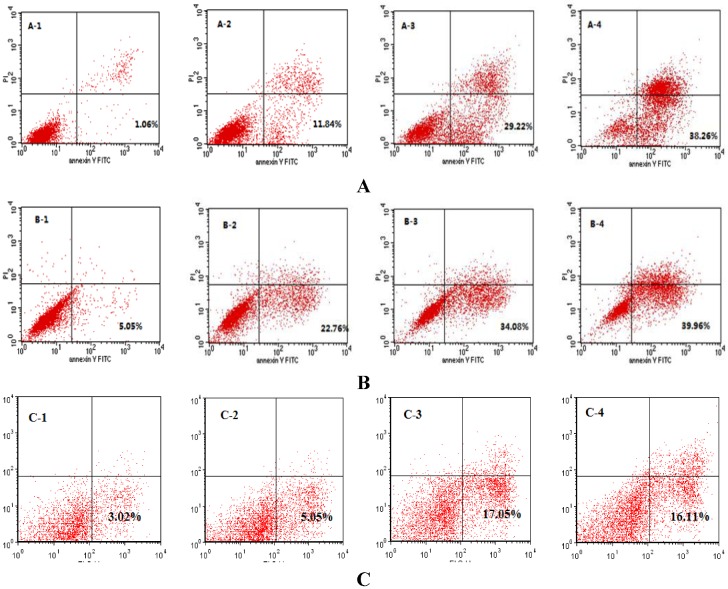
Flow cytometry analysis of DU-145 (**A**), PC-3 (**B**) and LNCaP (**C**) cells by double labeling with Annexin-V fluorescein isothiocyanate (FITC) and PI. Unfixed from control and treated cells were labeled with Annexin-V FITC and PI and then fixed and analyzed by flow-cytometry. Dual parameter dot plot of FITC—fluorescence (*x*-axis) *vs*. PI—fluorescence (*y*-axis) shows logarithmic intensity. Quadrants: lower left—the live cells. Lower right—the early apoptotic cells. Upper left—the necrotic cells, and upper right—late apoptotic cells. Percentages of early apoptotic cells were: (A-1) control cells: 1.06% ± 1.0%; (A-2) SIO (5 mg/mL): 11.84% ± 1.3%; (A-3) SIO (10 mg/mL): 29.22% ± 2.1%; (A-4) SIO (15 mg/mL): 38.26% ± 1.9%; (B-1) control cells: 5.05% ± 1.4%; (B-2) SIO (5 mg/mL): 22.76% ± 1.7%; (B-3) SIO (10 mg/mL): 34.08% ± 1.5%; (B-4) SIO (15 mg/mL): 39.96% ± 2.4%. One representative fluorescence activated cell sorting (FACS) assay of three independent experiments is presented. (C-1) control cells: 3.02% ± 2.4%; (C-2) SIO (5 mg/mL): 5.05% ± 1.3%; (C-3) SIO (10 mg/mL): 17.05% ± 2.5%; (C-4) SIO (15 mg/mL): 16.11% ± 1.4%. One representative FACS assay of three independent experiments is presented.

Apoptosis of DU-145, PC-3 and LNCaP cells was studied by flow cytometry analysis after treatment with SIO at concentrations of 5, 10, and 15 mg/mL for 24 h (Figure 3). The lower right quadrants represent the early apoptotic cells (Annexin V binding and propidium iodide (PI) negative). After a 24 h treatment with SIO, Annexin V and PI staining revealed that SIO increased apoptosis in DU-145 cells from 1.06% to 38.26% (Figure 3A) and in PC-3 cells (Figure 3B) from 5.05% to 39.96%. SIO also increased apoptosis in LNCaP (Figure 3C), but the number of early apoptotic cells was lower than DU-145 and PC-3.

### 2.4. Cell Cycle Analysis

**Figure 4:**
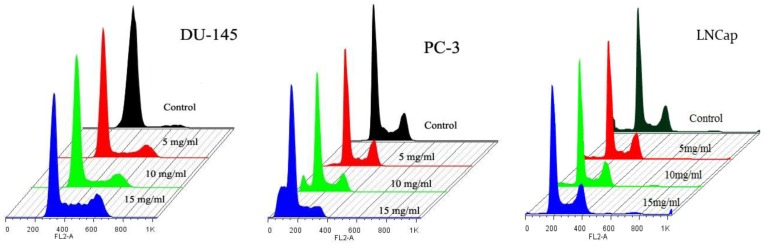
Effect of SIO on the cell cycle progression of DU-145, PC-3 and LNCaP cell lines. Cells were exposed at concentrations of 5, 10, and 15 mg/mL for 24 h. Flow cytometry was done to define the cell cycle distribution in comparison with untreated controls. The cell cycle profile is representative of three independent experiments.

Cell flow cytometry was used to determine the effect of SIO on the cell cycle progression. The cell cycle profile in Figure 4 is representative of three independent experiments including the three treatment groups in both cell lines. Table 1B shows that there are significant decreases in the numbers of cells from 23% ± 1.2% to 8% ± 2.0% in the S phase and from 26% ± 3.5% to 8% ± 2.3% in the G_2_/M phase in PC-3 after treatment with SIO at concentrations of 5, 10, and 15 mg/mL for 24 h. Moreover, there was a significant increase from 1% ± 1.3% to 24% ± 2.6% in the SubG_1_ phase and from 50% ± 3.5% to 60% ± 2.1% in the G_0_/G_1_ phase in PC-3. In contrast, there was a decreased proportion of DU-145 cells from 95% ± 2.1% to 65% ± 2.9% in the G_0_/G_1_ phase (Table 1A). The increased proportion of DU-145 cells was observed to be from 1% ± 3.8% to 19% ± 2.9% in the S phase and from 4% ± 4.7% to 16% ± 5.1% in the G_2_/M phase. As shown in Table 1C, SIO treatment induced strong S and G2/M phase cell cycle arrest in a dose-dependent manner in LNCaP.

**Table 1:** Percentage of DU-145 (**A**), PC-3 (**B**) and LNCaP (**C**) in Sub G_1_, G_0_/G_1_, S, and G_2_/M phase. Results represent mean ± SD of three independent experiments. Analysis of variance indicated a significant increase compared with the control (* *p* < 0.05).

**DU-145 (A)**							
		**SubG1**		**G0/G1**	**S**		**G2/M**
Control		0		95 ± 2.3	1 ± 3.4		4 ± 2.9
5 mg/mL		0		85 ± 2.2	8 ± 3.5		7 ± 2.6
10 mg/mL		0		80 ± 1.5	12 ± 3.9		8 ± 3.6
15 mg/mL		0		65 ± 1.9	19 ± 4.0		16 ± 2.4
**PC-3 (B)**							
		**SubG1**		**G0/G1**	**S**	**G2/M**	
Control		1 ± 4.3		50 ± 2.2	23 ± 2.3	26 ± 1.9	
5 mg/mL		3 ± 4.5		55 ± 3.0	20 ± 2.9	22 ± 2.1	
10 mg/mL		8 ± 3.9		58 ± 2.8	17 ± 3.4	17 ± 2.7	
15 mg/mL		24 ± 4.3		60 ± 1.9	8 ± 3.8	8 ± 3.5	
**LNCaP (C)**							
	**SubG1**		**G0/G1**		**S**		**G2/M**
Control	0		72 ± 1.2		3 ± 4.2		25 ± 4.5
5 mg/mL	0		70 ± 1.7		7 ± 3.5		23 ± 4.3
10 mg/mL	0		67 ± 2.1		7 ± 3.8		26 ± 2.9
15 mg/mL	0		62 ± 2.5		9 ± 4.1		29 ± 3.5

### 2.5. Western Blotting Results of Bcl-2, Bax and Caspase-3 in DU-145, PC-3 and LNCaP Cells Treated with SIO

**Figure 5:**
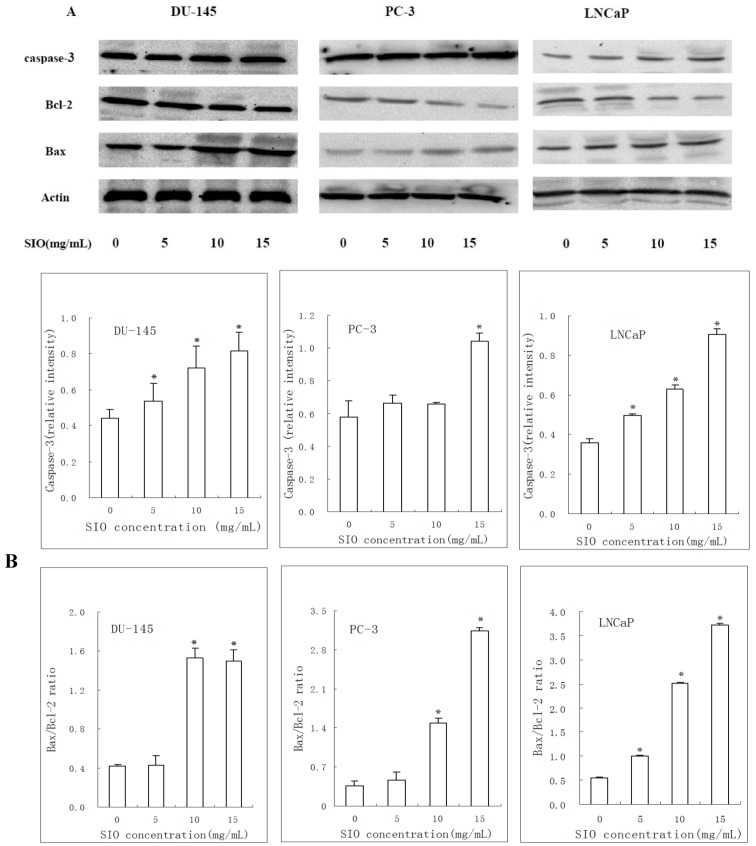
Expression of apoptosis-associated proteins in prostate cancer cells treated with SIO for 24 h. Representative blots shown from independent experiments are shown with identical results. Western blot was used to analyze whole cell lysates for Bax, Bcl-2, and caspase-3 expression upon SIO treatment, and equal amounts of the protein were loaded onto each lane (**A**). The relative intensity of caspase-3 and the Bax/Bcl-2 ratio were computed from three separate experiments using Quantity One (**B**). One representative immunoblot of three independent experiments is presented. * *p* < 0.05 versus control.

Based on the increased apoptosis of SIO- treated cells, we next examined whether caspase-3, Bcl-2 and Bax activities were changed by SIO treatment. Caspase-3 expression was significantly increased after SIO treatment (5, 10, and 15 mg/mL) for 24 h. Bax expression was significantly upregulated, whereas Bcl-2 expression was substantially downregulated (Figure 5A), and the ratio of Bax/Bcl-2 protein levels was also markedly increased (Figure 5B). These findings suggested that SIO induces apoptosis and might be involved in the regulation of the mitochondrion-mediated apoptosis pathway.

## 3. Discussion

The findings of the present study demonstrate the anticancer effects and associated mechanisms of SIO on prostate cancer cells in culture. Inhibitory effects of SIO were detected by CCK-8 assay *in vitro*, and SIO had strong inhibitory effects on DU-145, PC-3 and LNCaP cells. SIO inhibited DU-145, PC-3 and LNCaP cells in a dose- and time-dependent manner. 

Apoptosis is an important homeostatic mechanism that balances cell division and cell death and maintains the appropriate cell number in the body [20]. Many therapeutic peptides from marine animals, such as dolastatins-10 and 15, promote apoptosis in cancer cells. Moreover, some anticancer drugs cause the death of sensitive cells through the induction of apoptosis. In the present study, cell cycle analysis and Annexin V and PI double-staining assays revealed that SIO induced apoptosis in DU-145, PC-3 and LNCaP cells. DU-145 and PC-3 cells treated with SIO displayed the typical apoptotic cellular morphology. In addition, the percentage of early apoptotic DU-145, PC-3 and LNCaP cells was increased to 38.26%, 39.96% and 16.11%, respectively.

Further investigations focused on the mechanisms of SIO-induced apoptosis in prostate cancer cells. Apoptosis is a cell death process regulated in an orderly way by a series of signal cascades under specific situations. 

Two pathways are involved in cell apoptosis, extrinsic and intrinsic pathways [21]. Effector caspases are common to both the extrinsic and intrinsic pathways. Death receptors, through adaptor molecules, recruit initiator caspase-2, -8, or -10, while intrinsic death signals result in the activation of caspase-9 [22]. Initiator caspases cleave procaspases, and are thus able to activate effector caspases (caspase-3, -6 and -7) or to amplify the caspase cascade by increased activation of initiator caspases [23]. The SIO-induced apoptosis of DU-145 and LNCaP seemed to be related to the mitochondria-mediated pathway, based on the activation of caspase-3. But the data (PC-3 cells) for caspase-3 activation (at 5 and 10 mg/mL) does not correlate with apoptosis data. This investigation may suggest that the molecular mechanisms involved in SIO-induced apoptosis of PC-3 cells seem to be relevant to the intrinsic pathway. So we need further research to figure out the mechanisms, which may mainly contribute to the apoptosis of PC-3 cells.

One of the most important regulators of the mitochondrion-mediated pathway is the Bcl-2 protein family [24]. Members of this family, such as Bax, Bak, Bad, Bcl-Xs, Bid, Bik, Bim and Hrk, possess pro-apoptotic characteristics, while members such as Bcl-2, Bcl-XL, Bcl-W, Bfl-1 and Mcl-1 possess anti-apoptotic characteristics. The apoptosis-inducing effect is more dependent on the balance of Bcl-2 and Bax than on Bcl-2 quantity alone [25]. Typically, the ratio of Bcl-2 and Bax protein expression is used as an index of apoptosis [26]. We observed a remarkable up-regulation of Bax protein levels and a slight decrease in the Bcl-2 protein levels, leading to an increase of the Bax/Bcl-2 ratio in DU-145, PC-3 and LNCaP cells treated with SIO. Furthermore, SIO treatment induced strong S and G_2_/M phase cell cycle arrest in a dose-dependent manner in DU-145 and LNCaP. In contrast, SIO treatment induced strong Sub G_1_ and G_0_/G_1 _phase cell cycle arrest in a dose-dependent manner in PC-3. This observation is important, as the regulation of cell cycle in crucial in the growth and development of cancer.

In conclusion, these findings suggest that SIO, a tripeptide, has potent anticancer effects and causes cell apoptosis *in vitro*. SIO might be useful as a potential therapeutic agent against prostate cancer. Further examination of the mechanisms of the anticancer actions of SIO is in process; these studies may lead to new therapeutic options and improve the understanding of interactions between peptide compounds and gene regulation in human cancer. In addition, how SIO works, and the other mechanisms which may contribute to the changes of the cell cycle, and why the data (PC-3 cells) for caspase-3 activation (at 5 and 10 mg/mL) and for the Bax to Bcl-2 ratio (at 5 mg/mL) does not correlate with apoptosis data, should be the subject of further research. 

## 4. Experimental Section

### 4.1. Materials

**Figure 6:**
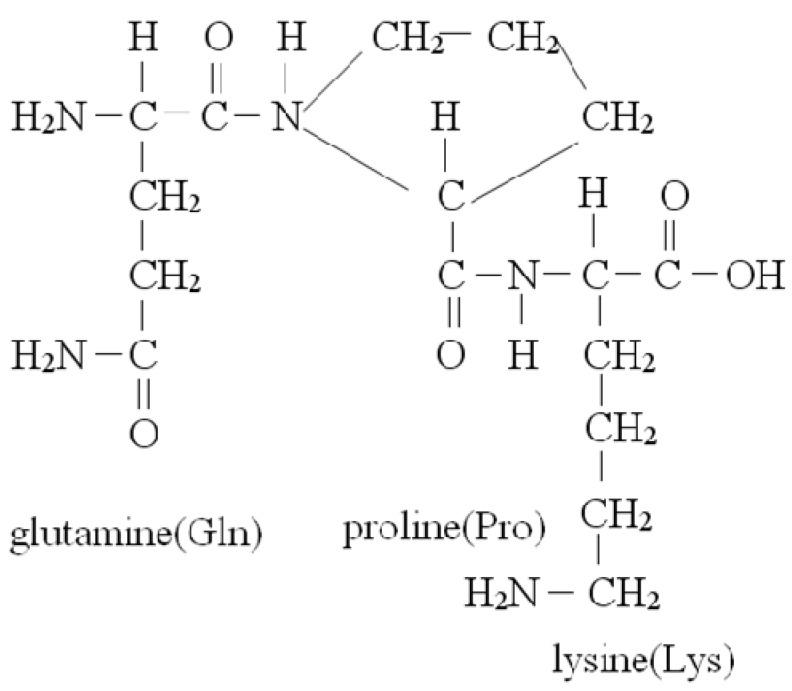
The amino-acid backbone of *Sepia* ink oligopeptides.

*Sepia* ink oligopeptides (*Sepia esculenta*) extracted from *Sepia* ink was hydrolyzed with trypsin to prepare peptides. Hydrolysates were isolated by ultrafiltration and purified using G-25 gel filtration. The purity of the *Sepia *ink oligopeptides was demonstrated by HPLC and its purity was above 95% [19]. Its sequence was identified as *N* Gln-Pro-Lys with a molecular mass of 343.4 Da. The amino-acid backbone of *Sepia* ink oligopeptides is shown in Figure 6. F-12 medium, fetal bovine serum (FBS), an annexin V-FITC apoptosis detection kit and CCK-8 assay were purchased from Technologies (Shanghai, China). Rabbit polyclonal antibodies against Bcl-2, Bax, caspase-3 and HRP-conjugated secondary antibodies were purchased from Cell Signaling (Beijing, China). 

### 4.2. DU-145. PC-3 and LNCaP Cells Culture

The hormone-independent DU-145, PC-3 and LNCaP prostate cancer cell lines were obtained from the China cell bank of the Institute of Biochemistry and Cell Biology. DU-145 and PC-3 cells were cultured in F-12 medium and LNCaP was cultured in 1640, supplemented with 10% Fetal Calf Serum (FCS) (1% penicillin/streptomycin; 10,000 U/mL penicillin G sodium, 10,000 mg/mL streptomycin sulfate). All cell lines were cultured at 37 °C in a humidified atmosphere in a 5% CO_2_ incubator. 

### 4.3. Anti-Proliferative Activity Using Cell Counting Kit-8(CCK-8) Assay

Cell proliferation after treatment with SIO was measured by CCK-8 assay. DU-145, PC-3 and LNCaP cells were seeded at a density of 1 × 10^4^ cells/100 µL/well in 96-well plates and allowed an overnight period for attachment. The medium was then removed and 200 µL of medium FCS was added, followed by SIO at a final concentration of 3, 5, 7, 10, 13 and 15 mg/mL. Cells grew under these conditions for 24, 48, and 72 h at 37 °C in a humidified atmosphere in a 5% CO_2_ incubator. After the designated time, CCK-8 was added to each well containing 200 µL of the culture medium and the oligopeptide mixture, and further incubated for 4–5 h at 37 °C. The amount of the formazan dye was measured at 450nm using a microplate reader. All experiments were performed in triplicate and repeated three times.

The percentage of inhibition of cell proliferation was calculated as follows: 


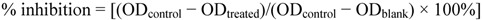


### 4.4. Morphologic Study with Fluorescence Microscope

The AO/EB staining (Acridine orange/Ethidium bromide) method was used to observe the apoptotic morphologic changes. DU-145 and PC-3 cells were suspended at a final concentration of 1 × 10^5^ cells/well and cultured above a 35 mm slip in six-well flat-bottomed plates and allowed to adhere to the bottom of the wells for 24 h before oligopeptide treatment. Cells were exposed to 5, 10, and 15 mg/mL doses of SIO for 24 h. F12 medium (10% FCS) was used as a control for the DU-145 and PC-3 cell lines. After the designed time, AO/EB mixture (25 µL, containing 100 µg/mL AO and 100 µg/mL EB in PBS (pH 7.4)) were added to the cells treated with SIO. Then the cells were observed under a fluorescence microscope.

### 4.5. Cell Apoptosis Analysis

Cell apoptosis detection was performed by fluorescence-activated cell sorting (FACS) analysis using a flow cytometer. The exposure of PS on the extracellular side of the cell membrane was quantified by annexin V-FITC/PI staining. DU-145, PC-3 and LNCaP cells were placed in six-well plates, and after 24 h of incubation, cells were treated with SIO for 24 h and then harvested. After centrifugation, cell pellets were washed twice with cold phosphate-buffered saline. Cells were then incubated with 5 μL of annexin V-FITC and 10 μL of PI at room temperature for 15 min in the dark. After incubation, 400 μL of 1× binding buffer was added to each tube. The cells were immediately analyzed by FACS Calibur flow cytometry (Becton Dickinson, USA).

### 4.6. Cell Cycle Analysis

Cell cycle perturbations were assessed using flow cytometry to measure the proportion of cells in the G1-, S- and G2/M-phases. Cell cycle perturbations induced by SIO were analyzed using propidium iodide (PI) DNA staining as described elsewhere. Approximately 1 × 10^5^ cells per well were plated in six-well plates and allowed to attach overnight. After 24 h of incubation, cells were treated with SIO for 24h and then collected, plated and fixed in ice-cold 70% ethanol for 4 h and stored at 4 °C until PI staining. Ethanol-suspended cells were then centrifuged at 1000 rpm for 5 min and washed twice in PBS to remove residual ethanol. Pellets were suspended in 1 mL of propidium iodide/RNase A reagent and incubated at 37 °C for 30 min. Cell cycle profiles were obtained using a BD FACScan Cell flow Cytometer (Becton Dickinson USA). 

### 4.7. Western Blot Analysis

Protein was extracted with RIPA buffer and quantified using the Bradford method. Equal amounts of protein were loaded to each well. After SDS-PAGE, proteins were transferred to a polyvinylidene difluoride (PVDF) membrane. The membrane was blocked with 10% non-immune serum for 2 h, and then incubated with primary antibody (Cell Signaling, Rabbit monoclonal antibody, 1:1000) at 4 °C overnight. After washing three times with TBST (Tris-buffered saline with 0.1% Tween-20) buffer, the membrane was incubated with the secondary antibody (goat-anti-rabbit horseradish peroxidase (HRP)-conjugated 1:3000) at room temperature for 2 h. The membranes were washed extensively with TBST. The intensity of the specific immunoreactive bands was detected by enhanced chemiluminescence (ECL), and quantified by densitometry and expressed as a ratio to actin. 

### 4.8. Statistical Analysis

Data are expressed as the mean ± standard deviation (SD). Statistical comparisons were performed using Student’s *t*-test, and differences between groups were considered significant at a value of <0.05. 

## 5. Conclusions

Our study demonstrates that SIO significantly inhibits the proliferation of DU-145, PC-3 and LNCaP cells by inducing apoptosis. Additionally, the expression ratio of Bax/Bcl-2 is increased after the treatment with SIO. Concurrently, the expression of caspase-3 is upregulated. These data support our hypothesis that SIO has anticarcinogenic properties and merits further investigation as a potential therapeutic agent.
